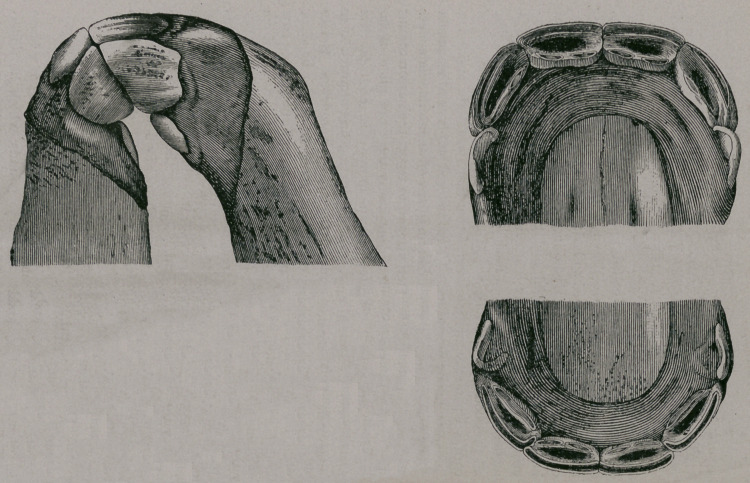# Age of the Horse, Ox, Dog, and Other Domesticated Animals

**Published:** 1890-07

**Authors:** R. S. Huidekoper

**Affiliations:** Veterinarian


### AGE OF THE HORSE, OX, DOG, AND OTHER DOMESTICATED ANIMALS.

By R. S. Huidekoper, M.D., Veterinarian.

[*Continued from page 346.*]

*First Period.—Eruption of the incisors of first dentition.* At the birth of the foal the incisors have not yet pierced the gums. The anterior border of the pincher and intermediate teeth can be seen under the mucous membrane, which is rendered paler than the surrounding tissues by their pressure. (Fig. 27.)

*About one week.*—The pincher teeth have generally appeared in from six to eight days; the upper teeth preceding the lower by twenty-four or forty-eight hours. At this age the teeth are of little importance; for the general aspect of the animal, its manner of walking, which is still unsteady, and the condition of its hairs shows that it is but a few days old.

*About one month.*—The intermediate teeth appear between thirty and forty days just as the interior border of the pincher teeth commence to be worn. (Fig. 28.)

*About three months.*—There are now four teeth in the upper and four teeth in the lower jaw; the pinchers have commenced to wear on their posterior borders; they are entirely free from the gum. (Fig. 29.)

*About four months.*—The incisive arch has become wider; the inferior intermediate teeth are free from the gum; their anterior border has commenced to be worn away toward their inner edge as it comes in contact with the corresponding superior teeth.

*About five months.*—The pincher teeth have pushed through the gums to the line of the neck of the teeth; the intermediate teeth are worn on their interior borders; the mucous membrane is often sensitive along the posterior border of these teeth on account of the corner teeth, which have commenced to push out. (Fig. 30.)

*About six months.*—The intermediate teeth have pushed further out; their posterior borders have come in contact with each other. In the region of the corner teeth the mucous membrane is puffy and congested; sometimes even at this age the anterior border of the corner teeth shows through the soft tissues.

*About eight to ten months.*—The anterior borders of the corner teeth are seen through the mucous membrane. The intermediate teeth are entirely through the gums to the level of their neck. The inferior incisive arch forms a regular half circle. (Fig. 31.) It is not important to be more precise at this age, as various causes influence and produce slight variations in the eruption of the teeth and in their leveling. Some animals are strong and vigorous while others are weak and feeble; some have been well fed while others have been nourished badly; and again, we find individual peculiarities of precocity and tardiness in the eruption of the teeth as in other evidences of development. At the outset, the foal only uses the milk of its mother; at this time there is little friction and using of the teeth, except from their simple position and contact with each other. As the foal gets older and commences to use fibrous and resisting food the incisors wear away more rapidly; there is always the most use in the pincher teeth. Moreover, during this first period of the life of the animal, while it is still with its mother, other conditions allow us to judge with sufficient accuracy as to the age of the animal which is not ready to be sold or removed until it has been weaned.

FIG. 27.

*At birth.*—(Fig. 27). The incisors are not yet out. The mucous membrane still covers the teeth which are about to appear. In front is seen under the gums the two pinchers, above and below. In profile are seen the intermediate teeth less developed than the pincher teeth. The jaws are rounded. The dental table shows on the side a little ridge formed by the anterior border of the pinchers with a less distinct elevation for the intermediate teeth on the side; the edge of the teeth near the symphysis of the jaw is higher that the outer edge, and comes through first.

FIG. 28.

*One month.*—(Fig. 28). In front the pincher teeth, which appeared during the first week, are in contact with each other ; their anterior face is striated with little gutters on the side ; the anterior border of the intermediate teeth is seen. In profile, the jaws are seen thicker ; the gums still cover part of the anterior border of the intermediate teeth. The dental tables are free from the pinchers. There is a slight use of both borders of the teeth ; below, the anterior border only has been worn. The membrane still covers the posterior border of the intermediate teeth and a portion of their dental cup.

FIG. 29.

*Three months.*—(Fig. 29). From in front, the pincher teeth are seen almost entirely free, though the gums still encircle the base of their free portion. The intermediate teeth are in contact by the internal portion of their anterior border. In profile, they are seen wider, thicker and less curved ; the intermediate teeth are not yet completely free. Their free borders are separated behind ; the dental tables are slightly used ; both borders of the intermediate teeth are slightly worn. The incisive arch increases in extent transversely,

FIG. 30.

*Five months.*—(Fig. 30). The jaws are thicker and the incisive arch is wider transversely. From in front the intermediate teeth are seen almost entirely free. In profile these teeth are in contact by the whole extent of their anterior border; the table of the pinchers are more worn, especially above. In each jaw the anterior border of the intermediate teeth is worn almost for its whole length; the posterior border is less worn. Below, is seen under the mucous membrane the internal edge of the corner teeth, which are preparing to pierce through. They form a little elevation of the mucous membranes placed immediately behind the internal border of the corresponding intermediate teeth.

FIG. 31.

*Ten months.*—(Fig. 31). These jaws come from a pony which was rather tardy in marking its age; nevertheless, they are useful plates from which to study the diverse characters of this period. The pinchers and intermediate teeth are entirely free from the gum; the anterior face of these teeth is polished, the little striations or canals are less visible; the intermediate teeth, which have been in contact for some months, are worn on both borders. The corner teeth are just appearing, but are not yet in contact with each other. The wearing of the pincher teeth is greater than that of the intermediate teeth.

## Figures and Tables

**Fig. 27. f1:**
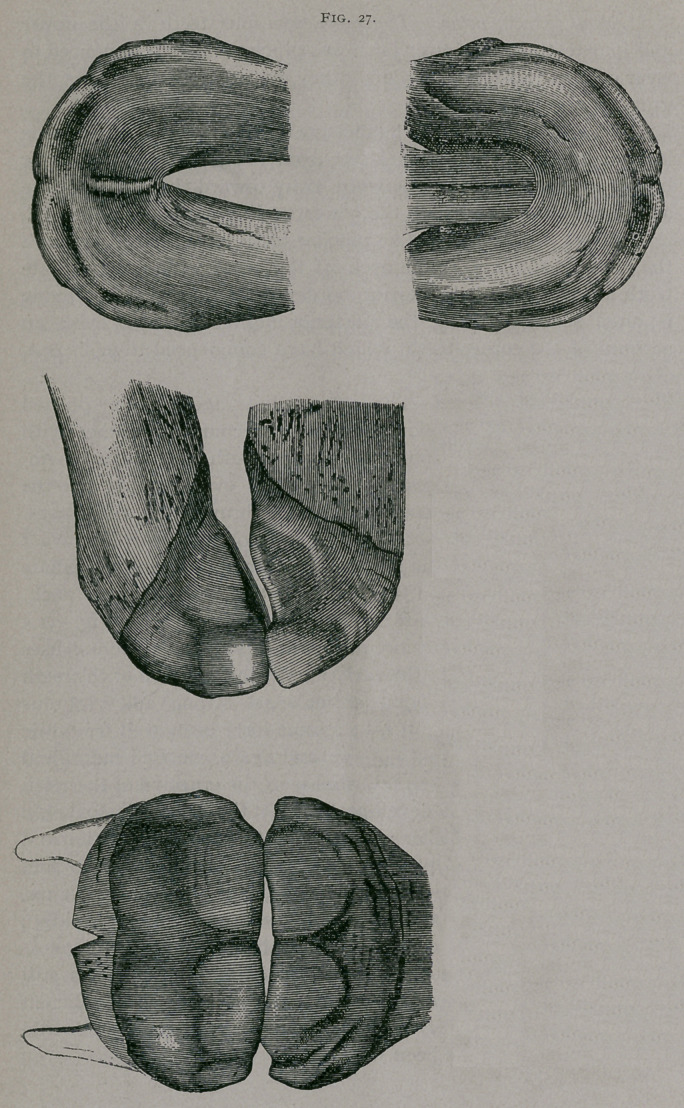


**Fig. 28. f2:**
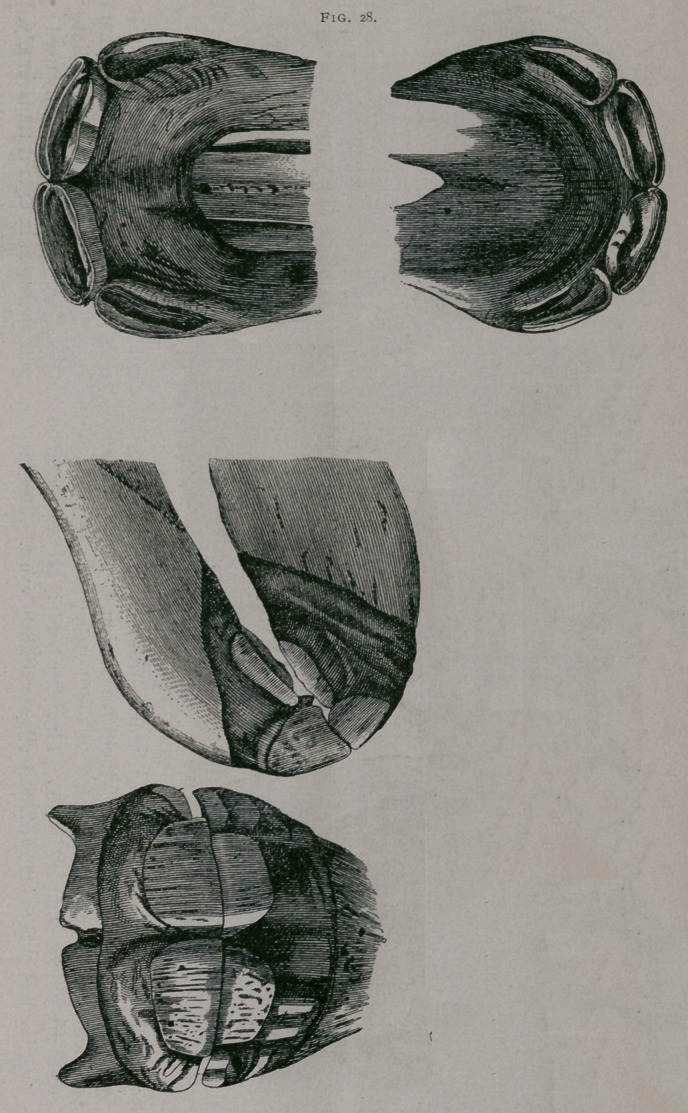


**Fig. 29. f3:**
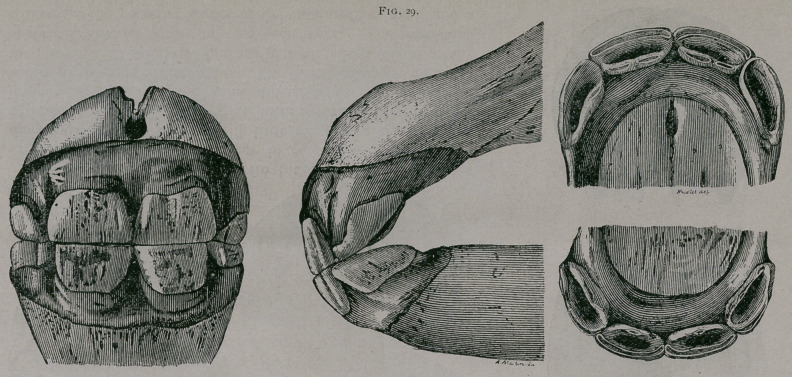


**Fig. 30. f4:**
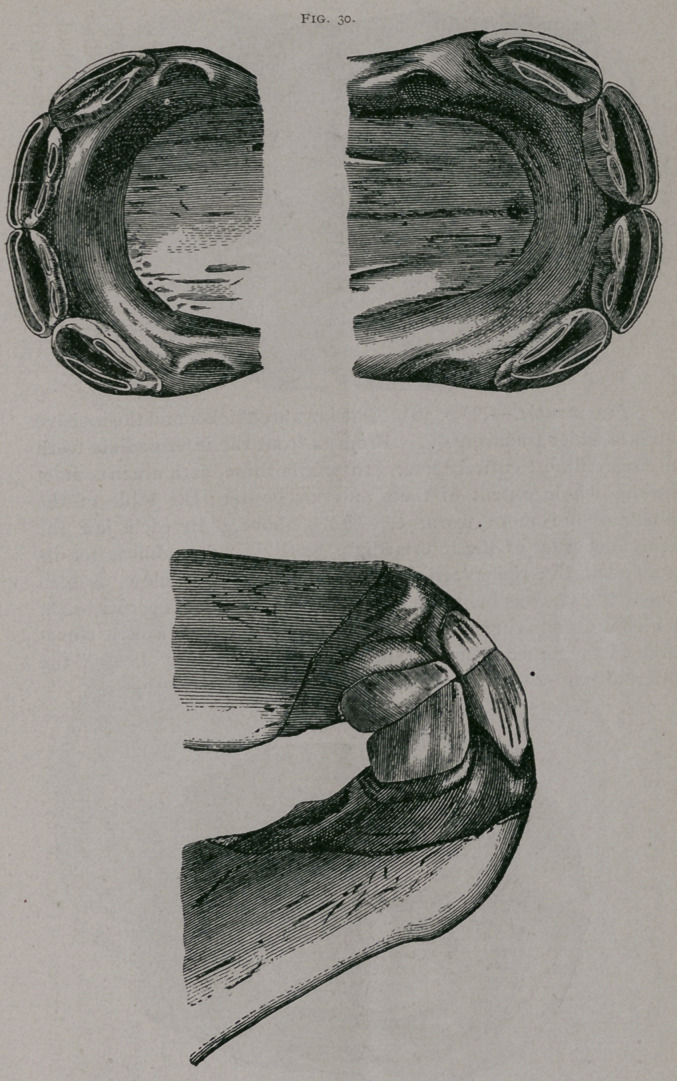


**Figure f5:**
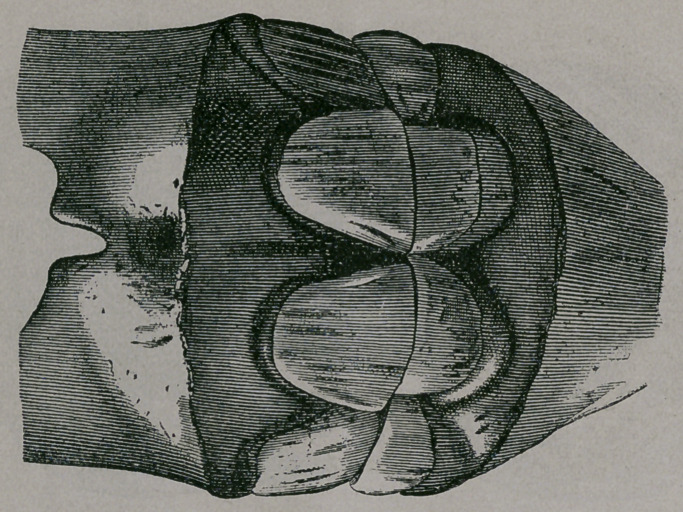


**Fig. 31. f6:**
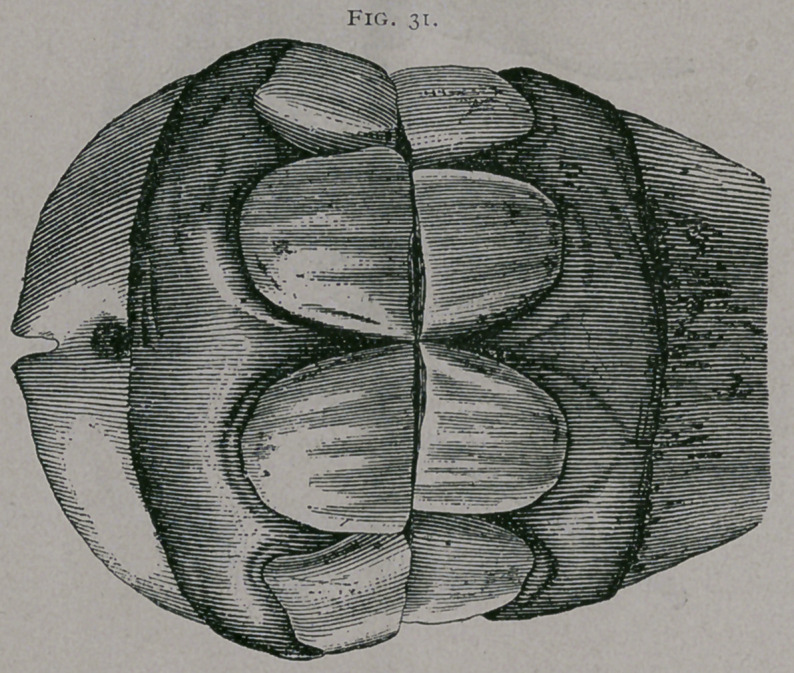


**Figure f7:**